# It's a Bird, It's a Plane, It's a Vein: Jugular Vein Phlebectasia in a Pediatric Patient With Tracheomalacia

**DOI:** 10.7759/cureus.42746

**Published:** 2023-07-31

**Authors:** Muhammad Afzal, Alaa Mohamed, Yakov Volkin

**Affiliations:** 1 Medical School, St. George's University School of Medicine, St. George's, GRD; 2 Pediatrics, Icahn School of Medicine at Mount Sinai, Elmhurst Hospital Center, Elmhurst, USA

**Keywords:** tracheo-esophageal fistula, otolaryngology, inpatient pediatrics, tracheomalacia, internal jugular phlebectasia

## Abstract

Jugular vein phlebectasia is seen in the first decade of life and carries a high chance of misdiagnosis as it can often be mistaken for other conditions observed in pediatric populations. High clinical suspicion along with radiological studies can help to confirm the diagnosis. Treatment is usually conservative, with surgery reserved for unique circumstances. This is the first case to be reported with concomitant tracheomalacia and a history of tracheoesophageal fistula repair in a pediatric patient with external jugular vein phlebectasia.

## Introduction

Jugular vein phlebectasia (JVP), also known as venous aneurysm, venous cyst, venous aneurysm, or venous dilatation, is a non-tortuous dilatation of the jugular vein [1-3]. Generally, it is a rare condition, with only 247 cases, including both adult and pediatric patients, identified in the most recent systematic review [3]. However, when JVP presents, it is seen in the first decade of life and is typically asymptomatic [1].

Differential diagnoses include other conditions observed in pediatric populations, such as laryngocele, cystic hygroma, and branchial cyst, for which JVP can often be mistaken [2]. First reported in 1928, much of the literature today still revolves around case reports and sparse literature reviews.

This case report aims to highlight a unique presentation of JVP and its diagnosis and management. Thus, clinicians can be more prepared when dealing with this entity and be aware of possible complications.

## Case presentation

A four-year-old male with a past medical history of tracheoesophageal fistula type-C repair on the second day of life, tracheomalacia, mild persistent asthma, small patent ductus arteriosus, and esophageal stenosis and pouch status-post rigid/flex esophagoscopy with steroid injection and balloon dilation presented to the pediatric emergency department (ED) with two months of constant cough, intermittent fever episodes with a maximum temperature (*T*_max_) of 103°F every two to three days, and one week of decreased appetite and lethargy. The patient’s most recent fever was one day prior to the presentation, with a *T*_max_ of 100.4°F treated with acetaminophen at home. Of note, the patient was recently treated four weeks ago for similar symptoms with a seven-day course of clindamycin for suspected pneumonia. Initial laboratory results were significant for an elevated white blood cell (WBC) count of (27.98 x 103)/µL with a right shift, with an absolute neutrophil count of 20.63 x 103)/µL. Radiological imaging included a chest X-ray, which showed right lower lobe consolidation, and subsequently, the patient was admitted for treatment of community-acquired pneumonia.

**Figure 1:**
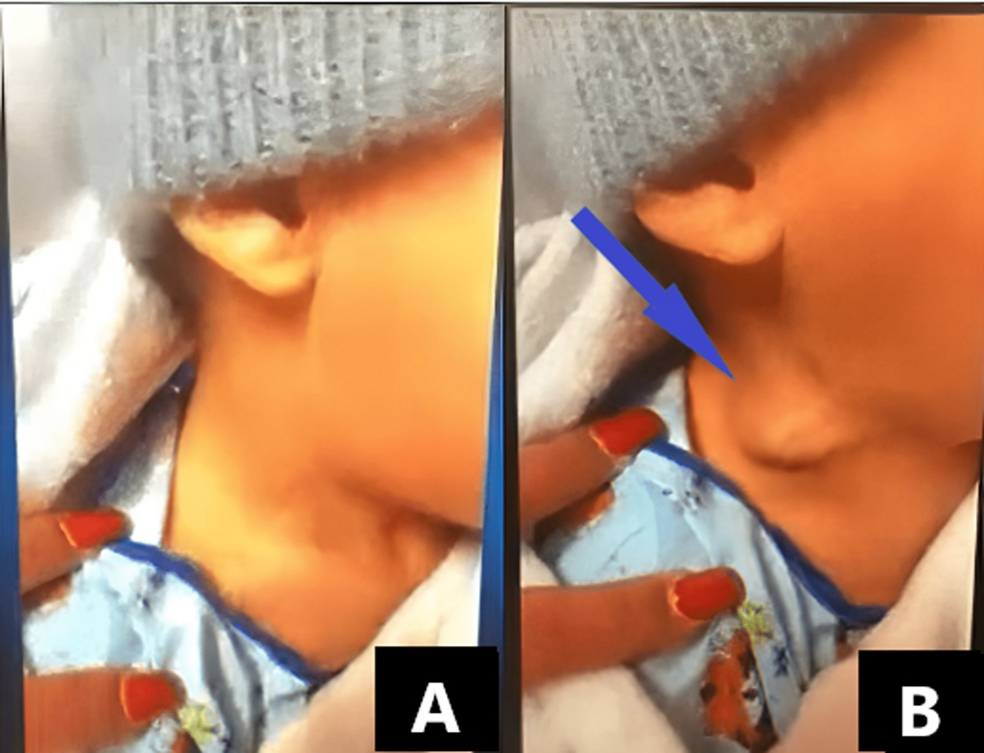
Right lateral view of the patient without (A) and with the Valsalva maneuver (B) demonstrating swelling in the jugular vein site (arrow).

On physical examination, the patient seemed mildly dehydrated with a barking cough but otherwise had no use of accessory muscles of respiration. As seen in Figure 1A, the initial neck examination did not reveal any abnormalities. However, upon coughing, a right-sided soft, compressible, ballotable, seemingly air-filled pocket was present. The mass appeared below the right sternocleidomastoid muscle and along the lateral aspect of the neck (Figure 1B). This was reproducible with coughing and Valsalva maneuvers such as straining. There were no associated pulsations or bruits, and the mass did not trans-illuminate. According to the patient’s parent, this mass was not present at any point prior to this current admission.

**Figure 2:**
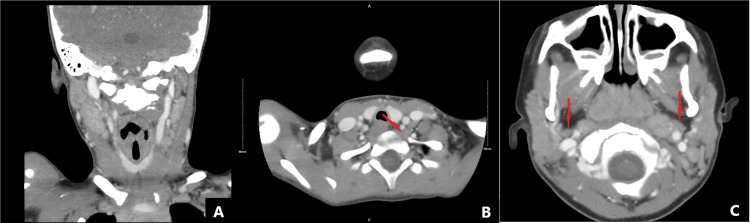
CT scan of the neck with contrast revealing (A) enlarged adenoids, (B) internal jugular lymphadenopathy (arrow), and retropharyngeal lymphadenopathy (arrows) without any masses. CT: computed tomography.

The otolaryngology (ENT) team was consulted and recommended a computed tomography (CT) scan of the neck with contrast. The CT scan showed enlarged adenoids, internal jugular lymphadenopathy, and retropharyngeal lymphadenopathy without any mass effects (Figure 2A-C). Additionally, the ENT team recommended the diagnosis of the external JVP. Due to the low risk of complications and absence of symptoms, no acute intervention or further work-up for this was recommended, and an outpatient follow-up was scheduled. He was treated with ampicillin-sulbactam and azithromycin while admitted. Upon discharge, the patient had a significantly improved cough and, subsequently, a less evident neck mass. No pathogens were isolated, and he was discharged with azithromycin. At a follow-up appointment four weeks later, no further neck swelling was noted, indicating resolution.

## Discussion

JVP is usually of unknown etiology, presenting as a compressible, soft mass in the neck during coughing, crying, and sneezing, which is reproducible via the Valsalva maneuver [1-4]. The lack of tortuosity and non-saccular and non-segmental nature differentiates it from varices and aneurysms, respectively [2]. It can arise in almost any cervicofacial vein but presents most commonly in the internal jugular, external jugular, anterior jugular, and superficial communicus in decreasing order of frequency [5]. There is a greater occurrence on the right side when compared to the left side or bilateral involvement. In a systematic review by Figueroa-Sanchez et al., left-sided predominance was only seen in 44 out of 247 total cases reported [3]. A proposed mechanism for this is that the right-sided internal jugular bulb is larger than the left side. Additionally, the right brachiocephalic vein is shorter and in direct continuity with the superior vena cava, unlike the left brachiocephalic vein [6]. Another hypothesis for right-sided preference is that the right innominate vein lies in contact with the apical pleura, allowing any increase in intrathoracic pressure to be communicated directly to the right internal jugular vein [7].

While the etiology is still unknown and debated, a possible hypothesis includes the loss of the normal connective tissue of the vein wall [8]. This can be from primary processes, such as congenital causes, or secondary to age-related degeneration of connective tissue in adults. Other causes may include mechanical compression, neck trauma, or gross anatomic abnormalities [9].

In the literature, phlebectasia of the external jugular vein has been reported very rarely and even less commonly in pediatric populations. In our case, the patient had a recent history of chronic cough, secondary to an infectious process. This could potentially have contributed to the discovery of right-sided phlebectasia. The constant stress from repeated bouts of coughing led to increased intrathoracic and intra-abdominal pressure, resulting in transient retrograde venous flow [10]. In fact, during coughing, the intrathoracic pressure increases from 100-250 mmHg, depending on the severity, to as high as 300 mmHg in adults [11].

Additionally, this patient also had a medical history of tracheomalacia, which is characterized by narrowing and increased collapsibility of the trachea, particularly during expiration [12]. It also presents commonly with other congenital defects, such as tracheoesophageal fistula, which was also seen in this case. In tracheomalacia, the weakening of the tracheal wall accelerates the airway narrowing that is seen during normal inspiration, leading to further increased intrathoracic pressure [12]. Phlebectasia of the external jugular vein could be more prone to arise in these situations.

The diagnosis of phlebectasia is made with an imaging study such as ultrasonography or color Doppler flow imaging because they are safe, non-invasive, and reproducible [5]. Doppler flow imaging can be used to show flow turbulence both with and without the Valsalva maneuver. It can also help to rule out other differential diagnoses of neck masses, such as laryngocele, mediastinal tumors, and branchial cysts, while remaining safe and low-cost [2,5]. In the majority of cases reported in a recent systematic review, ultrasound was employed as the primary study in 72% of cases [3]. CT scan is also sufficient in identifying the initial pathology, despite being utilized much less frequently [3,13]. However, Doppler flow imaging is preferred to visualize turbulence and luminal filling with and without Valsalva.

Most upper extremity and cervical venous aneurysms pose no significant risk to health or adverse outcomes [14]. This is in contrast to deep venous aneurysms, which are more commonly seen in the adult population and are more likely to contribute to thromboembolic events [14]. Treatment is usually conservative, which has been the approach in the majority of cases [3]. In fact, in the pediatric population of 102 reported cases, there have been no complications found from conservative management [3]. This makes it a safe choice for the majority of patients. However, surgical management can be considered in certain rare cases if there is an additional risk of complications. Surgical management is reserved for cosmetic reasons or risk of thrombus formation, pulmonary thromboembolism, or aneurysm rupture [14-16]. In our case, the patient was managed conservatively with treatment for pneumonia, and the phlebectasia did not require surgical intervention. Parental education was also provided regarding the pathology and possible complications that would have warranted a surgical intervention, as discussed before.

This case is unique from a clinical standpoint because the aneurysm was incidentally discovered at the time of presentation for pneumonia. The patient additionally had a history of tracheomalacia and past otolaryngological surgery for tracheoesophageal fistula repair. Past instrumentation and trauma to the area, along with excess collapsibility, might have created a situation that, when exacerbated by persistent coughing, could have led to the formation of this venous aneurysm. While many mechanisms have been proposed by various case reports, no definitive association has been drawn yet [3]. In clinical practice, the recognition of these factors is important to rule out other conditions since jugular venous phlebectasia is generally managed conservatively due to the low risk of complications [1,3].

## Conclusions

JVP should be considered in pediatric populations presenting with Valsalva-dependent neck swelling. In patients with pre-existing congenital or anatomical defects of the neck, a phlebectasia is more likely to arise if exacerbated by processes increasing intrathoracic pressure. Early recognition can prevent the use of unnecessary diagnostic imaging and increase patient comfort. The condition is usually self-limited and resolves spontaneously, with follow-up recommended for monitoring of changes or complications. Additional studies need to be performed to determine the impact of aneurysm size on spontaneous regression.
